# Implementation of Enhanced Recovery After Surgery Pathways in Gynecologic Oncology: Multidisciplinary Coordination, Nursing Roles, and Practical Challenges

**DOI:** 10.7759/cureus.115198

**Published:** 2026-08-25

**Authors:** Maria Bourazani, Chrysseida Maglari, Antonios Anagnostopoulos, Nikolaos Fyrfiris

**Affiliations:** 1 Department of Anaesthesiology, Hellenic Anticancer Institute, Saint Savvas Hospital, Athens, GRC; 2 Department of Gynecologic Oncology, Hellenic Anticancer Institute, Saint Savvas Hospital, Athens, GRC

**Keywords:** enhanced recovery after surgery, eras protocols, gynecologic oncology, implementation, multidisciplinary care, perioperative nursing, protocol adherence

## Abstract

Enhanced Recovery After Surgery (ERAS) pathways are evidence-based, multimodal approaches to perioperative care designed to reduce surgical stress, standardize clinical practice, and promote earlier functional recovery. In gynecologic oncology, their clinical benefits are well established; however, successful implementation remains challenging because of surgical complexity, variation in institutional resources, inconsistent adherence to protocol elements, and the need for coordinated multidisciplinary care.

This narrative review examines the practical implementation of ERAS pathways in gynecologic oncology, with emphasis on the translation of contemporary recommendations into routine clinical practice. Particular attention is given to multidisciplinary coordination, nursing roles, protocol adherence, patient engagement, organizational barriers, and long-term sustainability. Current evidence indicates that formal adoption of an ERAS pathway does not necessarily ensure consistent delivery of its individual components, while greater adherence is associated with improved postoperative outcomes.

Nursing professionals have a central role in ERAS implementation because they contribute to continuity of care across the perioperative pathway through patient education, symptom assessment, mobilization, nutritional support, recovery monitoring, and discharge preparation. Effective implementation also requires clearly defined professional responsibilities, structured communication, patient participation, and mechanisms for identifying clinically justified deviations from unwarranted variation in practice.

ERAS should therefore be regarded not as a static checklist but as a dynamic, multidisciplinary clinical system. Sustainable integration into gynecologic oncology practice depends on evidence-based standardization, appropriate individualization, active nursing and patient participation, and ongoing monitoring of adherence and outcomes.

## Introduction and background

Enhanced Recovery After Surgery (ERAS) represents a structured, evidence-based approach to perioperative care that integrates multiple interventions across the preoperative, intraoperative, and postoperative phases. ERAS pathways aim to attenuate the physiological stress response to surgery, preserve physiological function, reduce unwarranted variability in perioperative practice, and facilitate earlier functional recovery through coordinated multidisciplinary care [1,2].

In gynecologic oncology, ERAS is particularly relevant because patients may undergo complex pelvic or abdominal procedures in the context of malignancy, comorbidities, nutritional impairment, treatment-related vulnerability, and psychological burden. The ERAS Society has progressively updated its recommendations for this population, with the most recent guideline published in 2026 as the third major update of evidence-based recommendations for perioperative care in gynecologic oncology [2].

The clinical benefits of ERAS in gynecologic oncology are supported by systematic evidence. Bisch et al. reported that ERAS implementation was associated with a mean reduction in hospital length of stay of approximately 1.6 days, a 32% reduction in postoperative complications, and a 20% reduction in readmissions, without an increase in postoperative mortality [3]. An updated systematic review and meta-analysis subsequently confirmed reductions in length of stay and readmission across gynecologic surgical populations [4].

Despite this evidence, implementation remains heterogeneous. In an international survey of 454 clinicians from 62 countries, only 37% reported institutional ERAS implementation, while substantial variation persisted in adherence to several evidence-based pathway elements [5]. These findings illustrate the gap between the availability of ERAS recommendations and their consistent translation into routine clinical practice [2,5].

Successful implementation therefore requires more than the development of a written protocol. It depends on multidisciplinary coordination, clearly defined professional responsibilities, staff education, patient engagement, protocol adherence, and continuous evaluation of implementation processes and outcomes. Nursing professionals are particularly important because they provide continuity across perioperative phases and contribute directly to patient education, symptom assessment, mobilization, nutritional support, postoperative monitoring, and discharge preparation. 

The aim of this narrative review is to examine the practical implementation of ERAS pathways in gynecologic oncology, with particular emphasis on multidisciplinary coordination, nursing roles, patient engagement, protocol adherence, organizational barriers, and sustainability. Rather than repeating individual ERAS recommendations, the review focuses on the processes required to translate contemporary evidence into routine perioperative oncologic care.

Materials and methods

This article is a narrative review of the literature examining the implementation of ERAS pathways in gynecologic oncology, with particular emphasis on multidisciplinary coordination, nursing roles, protocol adherence, patient engagement, organizational barriers, and sustainability.

Literature search strategy

The literature search was initiated in 2018 as part of the background literature review conducted for the author's doctoral research and was progressively updated through 2024. For the purposes of the present narrative review, the search was further updated in August 2026 to incorporate newly published evidence, including the most recent ERAS Society recommendations. PubMed/MEDLINE, Scopus, and Google Scholar were searched using combinations of the terms "Enhanced Recovery After Surgery," "ERAS," "gynecologic oncology," "gynaecologic oncology," "gynecologic cancer," "perioperative care," "implementation," "protocol adherence," "compliance," "multidisciplinary team," "nursing," "patient education," "patient engagement," and "quality improvement." Boolean operators AND and OR were used where appropriate.

Priority was given to ERAS Society guidelines, systematic reviews and meta-analyses, randomized clinical trials, observational studies, implementation and quality-improvement studies, and qualitative research relevant to ERAS implementation in gynecologic oncology. Publications from 2016 to August 2026 were preferentially considered, while earlier seminal publications were included when necessary to provide historical or conceptual context. Reference lists of key guidelines and relevant reviews were also examined to identify additional relevant publications.

Studies focusing specifically on gynecologic oncology were prioritized. Evidence from benign gynecologic surgery or other surgical specialties was included selectively when it addressed implementation processes, multidisciplinary coordination, nursing practice, patient engagement, or organizational barriers for which gynecologic oncology-specific evidence was limited. Publications were considered eligible when they addressed ERAS implementation, clinical outcomes, multidisciplinary coordination, nursing practice, protocol adherence, patient engagement, or organizational barriers relevant to gynecologic oncology. Publications without direct relevance to these domains, non-human studies, and reports lacking sufficient clinical or implementation relevance were not retained for the final synthesis. A total of 34 publications were included in the final narrative synthesis. Because this was a narrative review, no formal risk-of-bias assessment or quantitative evidence synthesis was performed. The literature was organized thematically according to the principal domains of ERAS implementation addressed in this review.

## Review

Current ERAS framework in gynecologic oncology

ERAS pathways in gynecologic oncology integrate coordinated evidence-based interventions across the perioperative continuum to promote recovery and reduce unwarranted variation in care [1,2].

The most recent ERAS Society guideline for gynecologic oncology was published in 2026 and represents the third major update of recommendations for this population. It incorporates current evidence regarding preoperative optimization, nutritional management, anesthesia and analgesia, fluid therapy, maintenance of normothermia, thromboprophylaxis, early feeding, mobilization, and postoperative recovery [2].

A fundamental principle of ERAS is that its clinical effect derives from the combined application of multiple pathway elements rather than from any single intervention. ERAS should therefore be understood as an integrated perioperative system in which the effectiveness of individual components depends on their coordinated and consistent delivery throughout the surgical episode [1,2].

In gynecologic oncology, however, ERAS pathways must remain sufficiently flexible to accommodate differences in surgical extent, disease burden, comorbidities, intraoperative events, postoperative complications, and individual patient vulnerability. Deviations from specific pathway elements may therefore be appropriate when clinically justified, but they should represent individualized decision-making rather than routine departure from evidence-based practice.

The contemporary ERAS framework thus combines evidence-based standardization with individualized clinical judgment. Standardization aims to reduce unwarranted variation in perioperative care, while individualized decision-making ensures that the pathway remains appropriate for the oncologic and physiological needs of each patient.

From guideline to clinical practice: designing and adapting a local ERAS pathway

Translating ERAS recommendations into routine clinical practice requires conversion of guideline principles into a locally executable pathway. This includes defining what should occur during each phase of care, assigning responsibility for individual interventions, establishing documentation processes, and specifying how clinically justified deviations will be managed [2,6].

Local adaptation is necessary because institutions differ in staffing, infrastructure, available resources, documentation systems, discharge processes, and surgical case mix. However, adaptation should preserve the evidence-based core of ERAS rather than introduce unwarranted variation. The 2023 ERAS Society update emphasized that implementation barriers and poor adherence remain important despite the availability of established recommendations, highlighting the need for structured implementation strategies rather than guideline dissemination alone [6].

Implementation studies in gynecologic oncology demonstrate that structured institutional programs can improve both protocol adherence and clinical outcomes. In a prospective evaluation of patients with suspected or advanced ovarian cancer, ERAS implementation was assessed through protocol compliance and postoperative outcomes [7]. Similarly, Bisch et al. reported an increase in mean ERAS compliance from 56% to 77%, accompanied by reductions in hospital length of stay and in-hospital complications [8].

Implementation may be particularly challenging when an ERAS pathway is introduced across multiple clinical sites. Ackert et al. described the implementation of a gynecologic oncology ERAS protocol across a community hospital network and emphasized the need for coordinated participation from gynecologic oncology, anesthesiology, nursing, pharmacy, information technology, and quality-improvement teams. Their experience demonstrates that successful ERAS implementation requires organizational coordination and workflow redesign in addition to agreement on individual clinical components [9].

A central element of local pathway design is the explicit allocation of responsibilities across the perioperative continuum. Preoperative education, thromboprophylaxis, nutritional preparation, anesthetic management, postoperative analgesia, early feeding, mobilization, urinary catheter removal, and discharge preparation should not depend on informal expectations between disciplines. Instead, each component should be integrated into routine clinical workflows with clearly defined responsibilities and documentation processes [6,9]. This approach supports continuity across preoperative assessment, surgery, postoperative recovery, ward care, and discharge.

The importance of implementation fidelity is supported by evidence linking ERAS compliance with clinical outcomes. In an international prospective validation study, greater compliance with ERAS Society gynecologic/oncology guideline elements was associated with improved outcomes, including shorter hospital length of stay [10]. Similarly, another gynecologic oncology study found that compliance greater than 70% with modifiable ERAS elements was associated with a lower overall postoperative complication rate, suggesting that the effectiveness of an ERAS program depends not only on formal implementation but also on the consistency with which its components are delivered [11].

Nevertheless, implementation fidelity should not be interpreted as rigid adherence irrespective of clinical circumstances. Gynecologic oncology encompasses a broad spectrum of procedures, ranging from minimally invasive surgery to extensive cytoreductive procedures and pelvic exenteration. Evidence from pelvic exenteration suggests that standard gynecologic oncology ERAS pathways may require further optimization for patients undergoing exceptionally complex procedures [12]. Consequently, local ERAS pathways should distinguish between unwarranted variation in practice and clinically justified individualized deviation.

Taken together, current evidence supports a model in which local ERAS implementation combines evidence-based standardization with structured adaptation. The objective is not to reproduce guideline recommendations verbatim, but to translate them into an executable multidisciplinary pathway in which responsibilities, documentation, compliance monitoring, and mechanisms for clinical exceptions are clearly defined.

Multidisciplinary coordination and shared accountability

Effective ERAS implementation in gynecologic oncology requires coordinated contributions from the multidisciplinary perioperative team, including gynecologic oncologists, anesthesiologists, nurses, pharmacists, dietitians, and rehabilitation professionals. Rather than functioning as separate professional activities, these contributions should be integrated around shared recovery goals and clearly defined responsibilities [2,6].

The importance of multidisciplinary collaboration has been demonstrated in implementation studies. Ackert et al. described the introduction of a gynecologic oncology ERAS protocol across a 12-site hospital network using coordinated input from gynecologic oncology, anesthesiology, pharmacy, nursing, information technology, and quality-improvement teams. This collaborative approach supported the development of a comprehensive ERAS order set and the standardization of perioperative management across heterogeneous hospital settings [9]. The study illustrates that successful ERAS implementation requires both clinical agreement and organizational coordination.

Multidisciplinary coordination is particularly important at transitions between phases of care. Preoperative counseling should be consistent with intraoperative management and postoperative recovery goals. Anesthetic strategies should facilitate early awakening, symptom control, oral intake, and mobilization. Surgical decisions concerning drains, urinary catheters, and postoperative restrictions should be aligned with the recovery pathway, while ward-based care should reinforce rather than interrupt the intended ERAS trajectory. Inconsistent expectations between disciplines may lead to unnecessary variation and undermine protocol adherence [6,13].

Shared accountability is therefore an essential feature of ERAS governance. Responsibility for the pathway should not rest exclusively with a single professional group or an individual clinical champion. Instead, each discipline should have clearly defined responsibilities for specific ERAS elements while remaining accountable to common recovery objectives. This approach reduces the risk that clinically important components are omitted because responsibility is assumed to belong to another member of the team. The need for collective ownership is supported by broader implementation evidence. Nelson et al. evaluated the implementation of multiple ERAS pathways across a healthcare system and emphasized the importance of engaging multidisciplinary care teams in improving guideline adherence and surgical outcomes [14]. The study reinforces the concept that ERAS implementation should be integrated into organizational practice rather than depend on isolated individual initiatives.

Multidisciplinary coordination should also extend to the monitoring of pathway performance. Compliance with individual ERAS elements, postoperative outcomes, deviations from the pathway, and recurrent implementation barriers should be reviewed collectively. Audit and feedback allow the team to determine whether deviations reflect appropriate clinical individualization, unclear responsibilities, resource limitations, or persistence of traditional practices that are inconsistent with current recommendations. Audit of both process compliance and patient outcomes has been identified as a central characteristic of successful ERAS implementation [8,15].

Clear communication mechanisms are necessary to sustain shared accountability. Standardized order sets, structured clinical documentation, multidisciplinary meetings, and explicit escalation pathways can reduce ambiguity regarding recovery goals and responsibilities. However, these tools are effective only when embedded within a culture of collaborative decision-making and used to support, rather than replace, direct professional communication.

In gynecologic oncology, this collaborative model is especially important because patient trajectories may change rapidly according to surgical findings, complications, symptom burden, or functional recovery. Shared accountability does not imply rigid uniformity of care. Instead, it provides a framework within which individualized clinical decisions can be made transparently while maintaining alignment with the overall goals of ERAS.

The nursing role in ERAS implementation and continuity of care

Nursing professionals play a central role in the implementation and sustainability of ERAS pathways because many protocol components depend on continuous assessment, patient education, symptom management, mobilization, nutritional support, and coordination of care throughout the perioperative trajectory. Nurses therefore contribute not only to the delivery of individual ERAS elements but also to continuity between preoperative preparation, immediate postoperative recovery, ward-based care, and discharge planning [15-17].

The peri-anesthesia and immediate postoperative phases are particularly important because several determinants of early recovery are assessed and managed during this period, including pain, postoperative nausea and vomiting, respiratory function, hemodynamic stability, early detection of complications, readiness for oral intake, mobilization, and progression toward discharge criteria. A recent narrative review focusing specifically on peri-anesthesia nursing reported that ERAS protocols support greater consistency in clinical practice and are associated with improved postoperative recovery, pain management, earlier mobilization, and enhanced quality of care and patient safety. Peri-anesthesia nurses were identified as key contributors to patient monitoring, education, symptom management, and early recovery support [17].

The nursing contribution begins before surgery. Patient education is a core component of ERAS because patients are expected to participate actively in their recovery. Nurses can reinforce information regarding fasting and nutritional preparation, postoperative pain management, early oral intake, mobilization, respiratory exercises, and anticipated discharge milestones. Consistent information across the multidisciplinary team is particularly important because conflicting instructions may increase uncertainty and reduce adherence to recovery goals. Patient-experience research has also highlighted the importance of communication and symptom management throughout ERAS pathways and has identified nurses as being well positioned to support continuity of information across the perioperative period [18].

During postoperative recovery, nursing practice directly influences the delivery of several ERAS elements. Regular assessment of pain and postoperative nausea and vomiting, timely administration and evaluation of multimodal analgesia, support for early oral intake and mobilization, management and timely removal of invasive devices when clinically appropriate, and recognition of deviations from the expected recovery trajectory are all nursing-sensitive components of ERAS care. These activities contribute not only to patient comfort but also to functional recovery and prevention of complications [17].

Early mobilization illustrates the importance of nursing involvement particularly clearly. Prolonged postoperative bed rest is associated with muscle loss, pulmonary complications, venous thromboembolism, insulin resistance, and delayed functional recovery. However, successful early mobilization may be influenced by pain control, hemodynamic stability, patient motivation, staffing, clinical routines, and clear multidisciplinary expectations. Its implementation therefore requires active nursing assessment and coordination rather than a simple written instruction to mobilize the patient [19].

Nursing involvement extends beyond individual postoperative interventions and encompasses coordination across the entire perioperative pathway. A scoping review examining nursing interventions within ERAS identified contributions from the preoperative consultation through postoperative care and follow-up. These included patient preparation and education, pain management, support for early oral intake and mobilization, monitoring of recovery, and continuity of care following discharge [20]. The findings support the integration of nurses as active members of the ERAS multidisciplinary team and highlight their role in translating pathway recommendations into consistent clinical practice.

The effectiveness of nursing participation also depends on sustained education and familiarity with ERAS principles. In a single gynecological ward, Wickenbergh et al. found high agreement between patients and nursing staff regarding perceived adherence to ERAS principles, with further improvement following an educational session for nursing staff. These findings suggest that repeated education may contribute to maintaining adherence to ERAS recommendations in routine clinical practice [21].

The nursing role is also closely linked to patient safety. Continuous bedside presence places nurses in a position to identify early clinical deterioration, adverse effects of analgesic therapy, delayed recovery, inadequate oral intake, impaired mobility, and other factors that may require modification of the planned pathway. In this context, protocol adherence and clinical judgment are complementary rather than competing principles. Nurses contribute to ERAS fidelity while identifying situations in which individualized clinical intervention is required [17].

Nursing documentation also contributes to monitoring ERAS implementation because nurses routinely record key recovery milestones, including pain control, mobilization, oral intake, catheter removal, symptom burden, and discharge readiness. These data can help identify recurrent gaps between intended and delivered care and support multidisciplinary feedback and protocol refinement [15,22,23].

However, effective nursing participation requires adequate education, understanding of the rationale underlying protocol elements, clearly defined responsibilities, sufficient staffing, and organizational support. Without these conditions, ERAS activities may become fragmented or reduced to isolated tasks rather than functioning as components of an integrated recovery pathway. Strengthening nursing involvement in pathway design, implementation, monitoring, and evaluation may therefore improve continuity of care and protocol sustainability [17].

Overall, nursing professionals function as a critical link between ERAS recommendations and their practical implementation. Their contribution extends from patient preparation and postoperative surveillance to recovery support, patient engagement, discharge readiness, and quality monitoring. Recognizing nursing as an active implementation partner is therefore fundamental to achieving consistent and sustainable ERAS practice.

Analgesia, opioid-sparing strategies, and functional recovery

Effective postoperative analgesia is a core component of ERAS pathways because inadequate pain control can impair mobilization, oral intake, respiratory function, sleep, and participation in recovery. At the same time, excessive perioperative opioid exposure may contribute to sedation, postoperative nausea and vomiting, delayed gastrointestinal recovery, and impaired functional recovery. Contemporary ERAS pathways therefore favor multimodal analgesic strategies that provide adequate pain relief while reducing unnecessary opioid exposure [2].

In gynecologic oncology, opioid-sparing analgesia should be considered within the broader context of functional recovery rather than solely as a strategy for reducing opioid consumption. Earlier ERAS literature in this population incorporated multimodal opioid-sparing analgesia, including regional techniques when appropriate, as part of an integrated perioperative strategy designed to facilitate recovery and reduce adverse effects associated with traditional perioperative care [24].

More recent clinical evidence supports this approach. In a randomized clinical study of women undergoing major gynecologic oncologic surgery, an opioid-sparing multimodal anesthesia protocol incorporated within an ERAS pathway resulted in significantly lower postoperative pain scores and reduced requirements for rescue analgesia compared with conventional perioperative management. The ERAS group also demonstrated earlier oral intake, earlier gastrointestinal recovery, and earlier discontinuation of intravenous therapy, supporting the relationship between effective multimodal analgesia and broader postoperative recovery outcomes [25].

These findings were derived from the same randomized clinical study previously reported in a broader analysis of ERAS-related postoperative recovery outcomes [26]. That analysis demonstrated earlier mobilization, feeding, bowel recovery, discontinuation of intravenous therapy, and removal of urinary catheters and drains in the ERAS group. Taken together, the two reports describe complementary outcomes from the same patient cohort and reinforce the concept that analgesic effectiveness within ERAS should be evaluated according to its contribution to functional recovery rather than pain intensity alone.

Other gynecologic oncology studies have also reported reductions in opioid consumption following ERAS implementation. In minimally invasive gynecologic oncology surgery, ERAS programs have been associated with reduced opioid use, shorter hospitalization or increased same-day discharge, and favorable patient-centered outcomes [27]. In addition, implementation of a network-wide ERAS protocol for gynecologic oncology surgery identified reduction in opioid use as a principal outcome, demonstrating that opioid-sparing strategies can be incorporated into standardized perioperative pathways across different clinical settings [9].

However, opioid-sparing care should not be interpreted as complete opioid avoidance. The objective of multimodal analgesia is to balance adequate analgesia with minimization of treatment-related adverse effects while preserving the patient's ability to achieve functional recovery milestones. Individual patient characteristics, surgical approach, operative extent, contraindications to specific analgesic agents, and expected postoperative pain should therefore guide the selection and combination of analgesic modalities. The choice of individual analgesic components also requires evidence-based evaluation. For example, a randomized study in patients undergoing open gynecologic oncology surgery found no difference in postoperative opioid consumption between preoperative intravenous and oral acetaminophen when both were administered within an ERAS program. This suggests that more resource-intensive interventions should not automatically be assumed to provide additional clinical benefit [28].

Regional analgesic techniques may also have a role in selected patients, particularly following major open surgery. Evidence from gynecologic cancer surgery suggests that thoracic epidural analgesia can provide effective pain control when incorporated into an ERAS multimodal analgesic pathway. Selection of regional techniques should nevertheless be individualized according to surgical procedure, recovery objectives, institutional expertise, and potential effects on mobilization and hemodynamic stability [29].

From an implementation perspective, successful multimodal analgesia depends on coordination among anesthesiologists, surgeons, nurses, and pharmacists. A pharmacological protocol alone does not guarantee effective pain management. Regular nursing assessment of pain intensity, sedation, nausea and vomiting, functional capacity, and response to rescue analgesia is necessary to determine whether the analgesic strategy is facilitating or limiting recovery. Analgesic management should therefore remain integrated with mobilization, nutrition, gastrointestinal recovery, and other ERAS milestones.

Accordingly, the relevant endpoint in ERAS is not simply the lowest possible opioid dose or pain score. The broader objective is adequate analgesia with minimal treatment-related impairment, allowing early and safe restoration of function.

Protocol adherence and compliance as an implementation outcome

Protocol adherence is a central determinant of ERAS effectiveness. The presence of an institutional ERAS pathway does not necessarily indicate that its individual components are consistently delivered in clinical practice. Compliance should therefore be considered an implementation outcome in its own right and evaluated alongside traditional clinical outcomes such as complications, length of stay, readmission, and functional recovery.

Evidence from gynecologic oncology supports an association between greater ERAS compliance and improved postoperative outcomes. In an international prospective validation study, Wijk et al. demonstrated that higher adherence to ERAS Society gynecologic/oncology guideline elements was associated with shorter hospital length of stay and improved recovery outcomes [10].

Similarly, Pache et al. found that greater adherence to modifiable ERAS elements was associated with lower postoperative complication rates. Their findings support the importance of maximizing compliance with ERAS components, as the cumulative delivery of pathway elements appears to influence clinical outcomes [11].

This association has also been demonstrated specifically in ovarian cancer surgery. Sánchez-Iglesias et al. reported a significant relationship between increasing ERAS protocol compliance and decreasing hospital length of stay, with a particularly relevant effect among patients undergoing more complex procedures [30].

Implementation studies further demonstrate that compliance can improve substantially when ERAS is introduced through a structured institutional program. Bisch et al. evaluated 519 patients undergoing open surgery for presumed or confirmed gynecologic malignancy and found that mean compliance with ERAS elements increased from 56% before implementation to 77% after implementation. During the same period, median hospital length of stay decreased from four to three days, and complications before discharge decreased from 53.3% to 36.2% [8].

More recent multicenter evidence continues to reinforce the importance of compliance. Pergialiotis et al. evaluated adherence during early ERAS implementation across five tertiary gynecologic oncology institutions and identified substantial variability in uptake of individual protocol components. These findings indicate that formal adoption of a pathway does not ensure uniform implementation and that institutional and clinical factors may influence adherence [31].

Compliance assessment should therefore extend beyond reporting an overall percentage. Monitoring individual protocol elements can identify where implementation fails and help distinguish systematic barriers from clinically justified deviations. Recurrent non-adherence may reflect unclear responsibilities, insufficient education, inadequate documentation, resource limitations, or persistence of traditional perioperative practices. Conversely, deviations may be appropriate when dictated by surgical complexity, complications, comorbidities, or individual patient needs. ERAS fidelity should therefore not be interpreted as rigid adherence regardless of clinical context. The objective is to minimize unwarranted variation while preserving clinical judgment. Compliance data should be reviewed together with the reasons for non-adherence rather than interpreted solely as performance scores. From a quality-improvement perspective, monitoring adherence also creates an opportunity for multidisciplinary audit and feedback. Compliance with preoperative, intraoperative, and postoperative components can be evaluated alongside clinical outcomes to identify consistently delivered elements, components requiring targeted intervention, and areas in which pathway modification may be necessary. This approach transforms compliance measurement from a passive audit activity into an active mechanism for improving implementation.

Overall, the available evidence suggests that the effectiveness of ERAS in gynecologic oncology depends not only on whether a protocol has been formally introduced, but also on how reliably its components are delivered. Measuring adherence at both overall and component levels should therefore be incorporated into the governance and evaluation of ERAS programs.

Patient engagement, education, and shared recovery goals

Patient engagement is a fundamental component of ERAS because recovery depends not only on the actions of healthcare professionals but also on the patient's understanding of, and participation in, perioperative care. Patients are expected to engage actively in behaviors such as preoperative preparation, timely oral intake, mobilization, symptom reporting, and adherence to discharge and self-care instructions. Patient education should therefore be considered an implementation strategy rather than an optional supportive intervention [2,6].

Preoperative education should establish realistic expectations regarding the surgical pathway and postoperative recovery. Information should include anticipated pain-management strategies, nutritional progression, mobilization goals, removal of catheters and other devices, expected functional milestones, and discharge criteria. Structured and consistent communication can reduce uncertainty and help patients understand the rationale for early participation in recovery.

Qualitative evidence indicates that patients do not always fully understand ERAS even when they are treated within an ERAS pathway. Jenkins et al. identified gaps in patient knowledge among individuals undergoing gynecologic and gynecologic oncology surgery, highlighting opportunities to improve education and communication throughout the perioperative period [32]. Patient experience also depends on the consistency of information provided by the multidisciplinary team. A systematic review of qualitative studies found that patients valued clear information, symptom management, support, and continuity throughout the ERAS pathway, while inconsistent expectations or communication between healthcare professionals could negatively affect the recovery experience [18]. These findings reinforce the importance of shared recovery goals across disciplines.

Nursing professionals are particularly well positioned to reinforce patient engagement because of their continuous contact with patients across multiple phases of care. Nurses can assess understanding, clarify expectations, reinforce education, identify anxiety or misconceptions, and provide repeated guidance regarding mobilization, nutrition, pain control, and discharge preparation [17].

Patient engagement in gynecologic oncology requires particular sensitivity to the psychological and clinical context of cancer care. Patients may experience fear related to diagnosis, treatment outcomes, pain, postoperative dependence, and potential complications. Early discharge may also be perceived as premature or unsafe if the rationale and recovery criteria are not clearly explained. Education should therefore emphasize that ERAS aims to accelerate safe functional recovery rather than simply shorten hospitalization.

Shared decision-making and individualized communication are also important because patients differ in health literacy, language, social support, functional capacity, and psychological readiness. Standardized educational materials can improve consistency but should be supplemented by individualized discussion and confirmation of understanding. Written instructions alone should not be assumed to ensure meaningful engagement.

Patient participation also influences adherence to recovery goals. Early mobilization and oral intake cannot be achieved consistently without patient cooperation, but apparent non-adherence should not automatically be interpreted as lack of motivation. Pain, nausea, fatigue, dizziness, fear, inadequate explanation, or conflicting clinical instructions may all reduce participation. Assessment of these barriers should therefore precede conclusions about patient willingness to engage with the pathway.

Patient engagement should also be considered within ERAS evaluation. Patient-reported experience, understanding of recovery goals, satisfaction with education, and perceived preparedness for discharge may identify gaps that conventional clinical outcomes do not capture and can inform more patient-centered implementation.

Overall, effective ERAS implementation requires alignment between professional recommendations and patient understanding. Education, repeated communication, individualized support, and shared recovery goals are therefore essential components of a sustainable ERAS pathway in gynecologic oncology.

Organizational barriers and implementation challenges

Despite the established clinical benefits of ERAS, successful implementation remains inconsistent across gynecologic oncology settings. The availability of evidence-based recommendations does not ensure their routine application, and barriers may arise at individual, professional, organizational, and system levels, affecting adoption, adherence, and long-term sustainability [6,31].

One of the most persistent challenges is resistance to changing established perioperative practices. Traditional routines may remain embedded in clinical care even when newer evidence supports alternative approaches. The 2023 ERAS Society guideline specifically addressed this implementation gap and emphasized that several recommended practices continue to show poor adherence despite supporting evidence, indicating that guideline dissemination alone is insufficient to achieve practice change [6].

Variation in institutional resources also influences implementation. Staffing availability, access to dietetic or rehabilitation services, perioperative infrastructure, information technology support, and local documentation systems may determine whether individual ERAS elements can be delivered consistently. International survey data have demonstrated substantial variation in ERAS adoption across countries and institutions, with only 37% of respondents reporting implementation of an ERAS program at their institution [5].

Recent multicenter evidence confirms that adherence may remain variable even after formal ERAS adoption. Pergialiotis et al. evaluated implementation during the early adoption of ERAS across five tertiary gynecologic oncology institutions and found considerable variability in compliance with individual protocol components. Their findings support the concept that formal adoption should not be regarded as equivalent to effective integration into routine practice [31].

Education and professional understanding represent another important implementation domain. Healthcare professionals must understand not only which interventions are recommended, but also the rationale for their inclusion and their relationship to broader recovery goals. Inadequate knowledge, inconsistent training, or uncertainty regarding responsibilities may lead to selective implementation of individual ERAS elements rather than coordinated pathway delivery. This issue has also been documented from the nursing perspective. In a qualitative single-center study examining nurses' experiences of ERAS implementation, Akbuğa and Yılmaz identified barriers affecting compliance and routine clinical practice. Their findings emphasize the importance of nursing education, team communication, organizational support, and working conditions in translating ERAS recommendations into practice [33].

Importantly, implementation barriers are not always attributable to lack of awareness of the evidence. Knowledge of ERAS recommendations may coexist with practical constraints that prevent clinicians from changing established practice. Educational interventions alone are therefore unlikely to resolve barriers related to resources, workflow, staffing, professional roles, or organizational culture [34].

Specific ERAS components may also encounter distinct barriers. Early mobilization provides a useful example, as its implementation may be limited by inadequate staffing, insufficient education, pain, patient-related factors, uncertainty regarding mobilization criteria, or lack of appropriate clinical decision-support tools. A review of early mobilization within ERAS pathways identified lack of education and insufficient resources among modifiable barriers and suggested that education and clinical decision-making tools may improve implementation [19].

Sustainability represents an additional challenge. Initial enthusiasm following protocol introduction may decline over time, particularly when implementation depends on a limited number of clinical champions. Staff turnover, leadership changes, competing organizational priorities, and the gradual re-emergence of traditional practices may contribute to protocol drift. Sustainable implementation therefore requires mechanisms that embed ERAS into routine systems rather than relying primarily on individual motivation.

Leadership support is consequently important for maintaining ERAS pathways. Organizational leaders can facilitate implementation by supporting multidisciplinary governance, allocating resources, integrating ERAS elements into standardized documentation and order sets, and ensuring regular review of adherence and clinical outcomes. At the clinical level, visible leadership from surgeons, anesthesiologists, nurses, and other professional groups can reinforce shared expectations and help address barriers that cross disciplinary boundaries [6].

Implementation challenges should therefore be viewed as potentially modifiable characteristics of the healthcare system rather than as evidence that ERAS is unsuitable for routine practice. Identifying the specific causes of non-adherence at institutional and component levels allows interventions to be targeted more precisely. Education may address knowledge gaps, workflow redesign may clarify responsibilities, digital tools may improve documentation and prompting, and organizational investment may address resource limitations. This implementation-focused approach is more likely to support sustainable improvement than repeated dissemination of recommendations without corresponding system change.

Monitoring, auditing, and sustainability of ERAS implementation

ERAS implementation should not end with the introduction of a clinical pathway. Regular monitoring of protocol adherence and selected postoperative outcomes is essential to identify recurrent deviations, assess the consistency of pathway elements, and support long-term sustainability [6,8].

Adherence data should be interpreted alongside relevant clinical outcomes, including postoperative complications, length of stay, readmission, pain control, mobilization, and other recovery milestones. Multidisciplinary review and feedback can help distinguish clinically justified deviations from recurrent implementation failures and guide targeted modification of local practice [10].

The principal domains required to translate ERAS recommendations into sustainable clinical practice in gynecologic oncology are summarized in Table 1.

**Table 1:** Practical Framework for ERAS Implementation in Gynecologic Oncology Key domains, professional responsibilities, nursing contributions, and monitoring indicators supporting sustainable ERAS implementation in gynecologic oncology. Abbreviations: ERAS, Enhanced Recovery After Surgery; PONV, postoperative nausea and vomiting.

Implementation domain	Key actions	Main professionals involved	Nursing contribution	Suggested monitoring indicators
Local pathway design	Adapt ERAS recommendations to local workflow while preserving core evidence-based elements	Gynecologic oncologists, anesthesiologists, nurses, pharmacy, quality team	Identify nursing-sensitive processes, workflow feasibility, documentation needs	Defined responsibilities; standardized pathway; documentation completeness
Preoperative preparation and patient engagement	Provide structured education and establish recovery expectations	Surgeons, anesthesiologists, nurses, dietitians	Assess understanding, reinforce instructions, explain recovery milestones	Education completed; patient understanding; preparedness for surgery
Coordinated perioperative care	Align anesthetic, surgical, analgesic, nutritional, mobilization, and device-management strategies	Multidisciplinary perioperative team	Symptom assessment, mobilization, oral intake support, recovery monitoring	Pain/PONV control; time to mobilization; oral intake; device removal
Protocol adherence and individualized deviation	Monitor delivery of ERAS elements and distinguish justified deviation from unwarranted variation	ERAS team and clinical leads	Document adherence and reasons for deviations	Overall compliance; component-level compliance; documented deviations
Multidisciplinary communication	Maintain consistent goals and clarify responsibilities across transitions of care	Entire multidisciplinary team	Ensure continuity between perioperative phases and communicate recovery status	Missed elements; communication-related deviations
Audit and sustainability	Review adherence and outcomes and refine local practice	ERAS team, leadership, quality team	Contribute nursing-sensitive recovery data and identify recurrent barriers	Complications; length of stay; readmission; adherence trends; recovery milestones

Discussion

The evidence reviewed indicates that ERAS is now a well-established framework for perioperative care in gynecologic oncology. The principal contemporary challenge is therefore not whether ERAS is clinically beneficial, but whether evidence-based recommendations can be translated into consistent, multidisciplinary, and sustainable clinical practice. This distinction is particularly important because the 2023 ERAS Society update identified persistent implementation barriers and poor adherence to several recommended practices, while the 2026 update further consolidated the current evidence base for perioperative care in gynecologic oncology [2,6].

The findings of this review suggest that successful implementation requires ERAS to be understood as an integrated perioperative system rather than a collection of isolated interventions. The effectiveness of individual components depends partly on their coordination with other elements of care and on their reliable delivery across the preoperative, intraoperative, and postoperative phases. System-wide implementation studies support this interpretation. Bisch et al. demonstrated that structured ERAS implementation increased overall protocol compliance and was accompanied by reductions in hospital length of stay and in-hospital complications, linking organizational implementation with clinically meaningful outcomes [8].

Protocol adherence therefore represents an important implementation variable. Formal adoption of an ERAS pathway does not guarantee consistent delivery of its recommended components, while greater compliance appears to be clinically meaningful. In ovarian cancer surgery, increasing adherence to ERAS recommendations was associated with shorter hospital length of stay, reinforcing the importance of monitoring how reliably the pathway is implemented rather than simply whether it is formally present [30]. Compliance assessment should therefore form part of routine ERAS governance rather than remain solely a research outcome. At the same time, adherence should not be interpreted as rigid conformity. Gynecologic oncology encompasses substantial variation in disease burden, surgical complexity, comorbidities, and postoperative risk, and clinically justified deviations may therefore be necessary. The relevant distinction is between appropriate individualization and unwarranted variation arising from inconsistent practice, inadequate communication, or failure to implement evidence-based recommendations. ERAS programs should therefore reduce avoidable variability while preserving individualized clinical decision-making.

Multidisciplinary coordination is central to achieving this balance. ERAS implementation involves interdependent contributions from surgeons, anesthesiologists, nurses, pharmacists, rehabilitation professionals, dietitians, and other members of the perioperative team. Implementation across a community hospital network has shown that coordinated involvement of clinical disciplines together with pharmacy, information technology, and quality-improvement teams can support standardized ERAS delivery across heterogeneous settings [9]. Shared accountability is therefore preferable to assigning responsibility for ERAS to a single professional group or clinical champion.

Within this multidisciplinary structure, nursing has a particularly important implementation role. Many ERAS components are translated into practice through continuous nursing assessment and care, including symptom monitoring, pain management, early oral intake, mobilization, patient education, management of invasive devices, and discharge preparation. Nurses are therefore positioned not only to support pathway progression but also to recognize early deviations from expected recovery. Their contribution should be understood as part of pathway coordination, surveillance, continuity, and patient safety rather than merely as execution of prescribed interventions.

This role is especially evident when considering functional recovery. Postoperative analgesia should not be assessed solely by pain scores or opioid consumption, but by its ability to facilitate mobilization, oral intake, gastrointestinal recovery, sleep, and participation in care. Evidence from gynecologic oncology demonstrates that opioid-sparing multimodal anesthesia can improve several of these recovery outcomes, supporting a broader interpretation of analgesic effectiveness within ERAS [25].

Patient engagement represents another critical dimension of implementation. ERAS requires active participation in recovery, yet qualitative evidence indicates that patients undergoing ERAS-guided gynecologic and gynecologic oncology surgery may have incomplete knowledge of the pathway and its objectives [32]. Providing information should therefore not be assumed to ensure understanding. Structured education, repeated communication, and confirmation of patient understanding should be integrated into pathway implementation, particularly in oncology, where anxiety, uncertainty, symptom burden, and concerns about early discharge may influence participation in recovery goals.

Implementation barriers are also multidimensional. Limited adherence may reflect resource constraints, staffing, entrenched clinical routines, insufficient education, fragmented communication, inconsistent professional expectations, or organizational systems that do not support the pathway. The persistence of these barriers despite repeated publication of ERAS recommendations demonstrates that dissemination of evidence alone is insufficient to change practice [6]. Implementation strategies should therefore address local determinants of non-adherence rather than assume that guideline availability will automatically produce behavioral or organizational change.

Finally, ERAS implementation should be regarded as an ongoing process rather than a one-time intervention. Protocol introduction should be followed by monitoring of adherence and selected clinical outcomes, multidisciplinary review of recurrent deviations, and targeted refinement of local practice. Audit and feedback can help identify protocol drift and distinguish clinically appropriate deviations from systematic implementation failures [6].

Taken together, the evidence supports a model of ERAS implementation in gynecologic oncology based on four interdependent elements: evidence-based standardization, multidisciplinary ownership, active nursing and patient participation, and continuous monitoring of adherence and outcomes. The effectiveness of ERAS therefore depends not only on the quality of individual recommendations but also on the capacity of healthcare organizations to integrate them reliably into everyday perioperative practice.

Limitations

This narrative review has several limitations. Although the literature search included PubMed/MEDLINE, Scopus, and Google Scholar, the review was not conducted systematically and did not include a formal risk-of-bias assessment or quantitative synthesis, which may introduce selection bias. The available evidence is also heterogeneous with respect to surgical approach, oncologic diagnosis, institutional setting, ERAS components, implementation strategies, and outcome definitions. In addition, some of the evidence concerning nursing practice, patient engagement, and organizational barriers derives from broader gynecologic or perioperative populations rather than exclusively from gynecologic oncology, which may limit the generalizability of the findings across different clinical settings and patient populations.

## Conclusions

Enhanced Recovery After Surgery has become an evidence-based standard of perioperative care in gynecologic oncology, but its effectiveness depends on the consistent translation of recommendations into routine clinical practice. Successful implementation requires multidisciplinary coordination, clearly defined professional responsibilities, meaningful nursing involvement, patient engagement, and sustained adherence to evidence-based pathway elements, while allowing clinically justified individualization according to surgical complexity and patient needs.

Nursing professionals play a central role in maintaining continuity across the perioperative pathway through patient education, symptom assessment, mobilization, nutritional support, recovery monitoring, and discharge preparation. ERAS should therefore be regarded as a dynamic clinical system rather than a static checklist, with ongoing monitoring of adherence and outcomes, multidisciplinary review, and targeted refinement of local practice supporting long-term sustainability in gynecologic oncology.
